# Primary Histiocytic Sarcoma of the Breast: A Case Report and Review of the Literature

**DOI:** 10.7759/cureus.59677

**Published:** 2024-05-05

**Authors:** Hind Althomali, Haneen Al-Maghrabi, Nora Trabulsi, Jaudah Al-Maghrabi

**Affiliations:** 1 Department of Pathology and Laboratory Medicine, King Abdulaziz University Hospital, Jeddah, SAU; 2 Department of Pathology and Laboratory Medicine, King Faisal Specialist Hospital and Research Center, Jeddah, SAU; 3 Department of Surgery, King Abdulaziz University Hospital, Jeddah, SAU; 4 Department of Pathology, King Abdulaziz University Faculty of Medicine, Jeddah, SAU

**Keywords:** cancer, immunohistochemistry, malignancy, histiocytic sarcoma, breast

## Abstract

Histiocytic sarcoma (HS) is a rare cancerous tumor that originates from fully developed histiocytes. It is most often identified by the presence of certain proteins such as the cluster of differentiation (CD) 68, CD163, or lysozyme. HS has been recorded in different sites outside of the lymph nodes such as the gastrointestinal tract, nasal cavities, skin, and bone marrow. Because HS shares similar clinical features with other forms of malignant diseases, diagnosing it becomes incredibly challenging. We report a case of a 40-year-old female who presented with a breast mass for one year. A preliminary diagnosis of a phyllodes tumor was made. However, the morphology along with the immunophenotype picture was diagnostic for HS. Microscopic features showed a well-defined neoplastic growth arranged in sheets and fascicles. Diffuse immunoreactivity was seen for CD45, CD4, CD68, CD163, and vimentin. We present the important histopathological and immunohistochemical characteristics of the tumor in this case.

## Introduction

Histiocytic sarcoma (HS) is an extremely uncommon and highly aggressive disorder of histiocytes, where malignant cells proliferate and bear a resemblance to histiocytes [1]. HS accounts for less than 1% of all malignancies in the hematopoietic system in terms of incidence. Most cases of HS are found in people aged 40-50 years, with an average age of 46 years old, and a slightly higher prevalence in males [2]. Frequently, HS presents itself in non-lymph node areas such as the skin, nasal cavity, and gastrointestinal tract [3]. Clinical features of the disease can involve variable clinical presentations ranging from localized disease to multi-organ involvement. HS often exhibits non-specific symptomatology including fever, anorexia, and asthenia along with hepatosplenomegaly, lymphadenopathy, and pancytopenia [4]. HS can manifest either on its own or alongside other hematolymphoid disorders such as multiple myeloma, non-Hodgkin lymphoma, chronic lymphocytic leukemia, and mantle cell lymphoma [5]. The definitive diagnosis of HS should rely on the evaluation of cytomorphology and immunophenotypical characteristics through pathological examination. The disease is so rare and rapidly progressive that there is very little clinical data available. This lack of information has led to the absence of a standardized treatment protocol for HS. Treatment modality is usually based on the severity and localization of the disease. Surgery is the preferred option when the disease is localized along with adjuvant therapy if required. In the case of multi-organ involvement, chemotherapy is usually preferred [6]. Clinicians face a notable challenge in managing HS due to its aggressive behavior, unfavorable prognosis, and absence of definitive data on a preferred standard treatment approach.

## Case presentation

A 40-year-old woman arrived at our hospital with a substantial and continuously expanding tumor in her right breast. No significant medical or surgical intervention was found in her previous medical records. There was no family history of cancer. Previously, she presented around a year ago to our hospital with the findings of a right breast mass which was subsequently identified as fibroadenoma after an ultrasound assessment. A close follow-up was recommended but unfortunately, the patient did not attend her scheduled follow-up appointment and could not be reached for further contact. The mass then started to grow so she sought medical opinion at our center.

**Figure 1:**
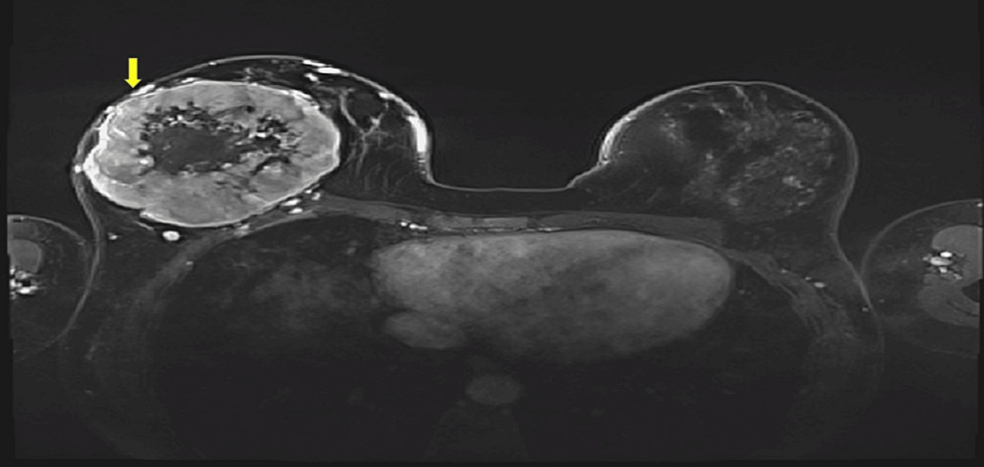
MRI reveals an uneven enhancement pattern and lack of enhancement in the central region.

Upon physical examination, the whole right breast appeared to be replaced with a huge mass which was associated with dilated veins. The mass was fixed to the overlying skin and nipple areola complex with no clear chest wall invasion. A bilateral mammogram and ultrasound showed a huge solid mass with intrinsic cystic changes occupying almost the entire right breast measuring 10 x 8 cm. These findings were suggestive of a large malignant phyllodes tumor, for which a core biopsy and bilateral breast magnetic resonance imaging (MRI) were recommended to confirm the diagnosis and assess the extent of the disease. Bilateral breast MRI showed a larger right breast compared to the left breast which was occupied by a huge well-circumscribed lobulated mass appearing as hypointense on T1. Contrast examination, on subtracted images, showed heterogeneous enhancement of the large part of the mass with non-enhancement of the central area at the site of necrosis (Figure 1).

Ultrasound-guided core biopsy was performed and sent for pathology evaluation. Histopathological results of the core biopsy showed malignant spindle cell neoplasm. Differential diagnoses included malignant phyllodes, metaplastic carcinoma, and primary sarcoma of the breast. Complete staging workup including computerized tomography (CT) of the chest, abdomen, and pelvis was negative. After discussion at the multidisciplinary tumor board (MDTB), the decision was to proceed with mastectomy and immediate reconstruction using a latissimus dorsi myocutaneous flap.

**Figure 2:**
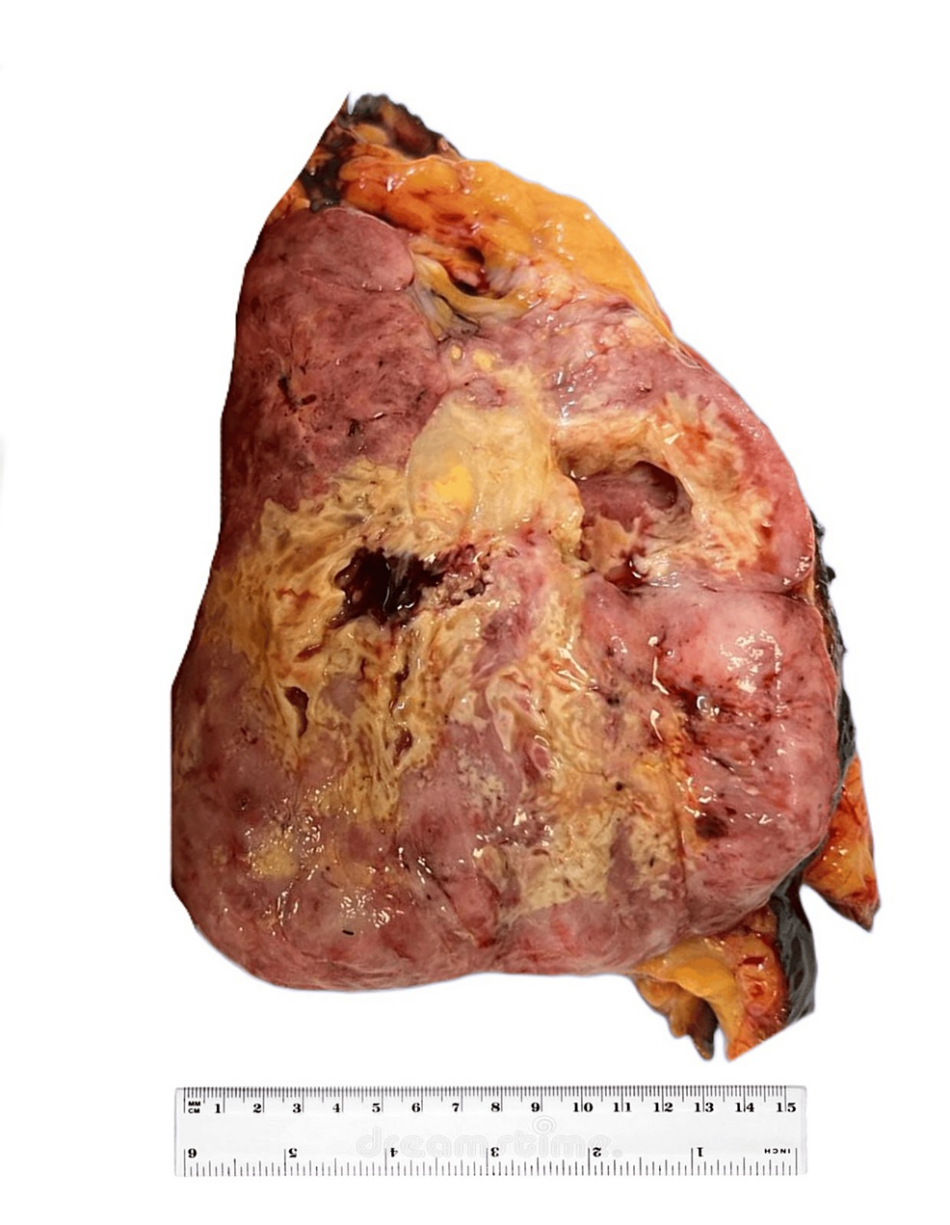
Gross examination of right mastectomy specimen. The round solid mass is approximately 15 x 13 x 8 cm. Area of necrosis is noted.

**Figure 3:**
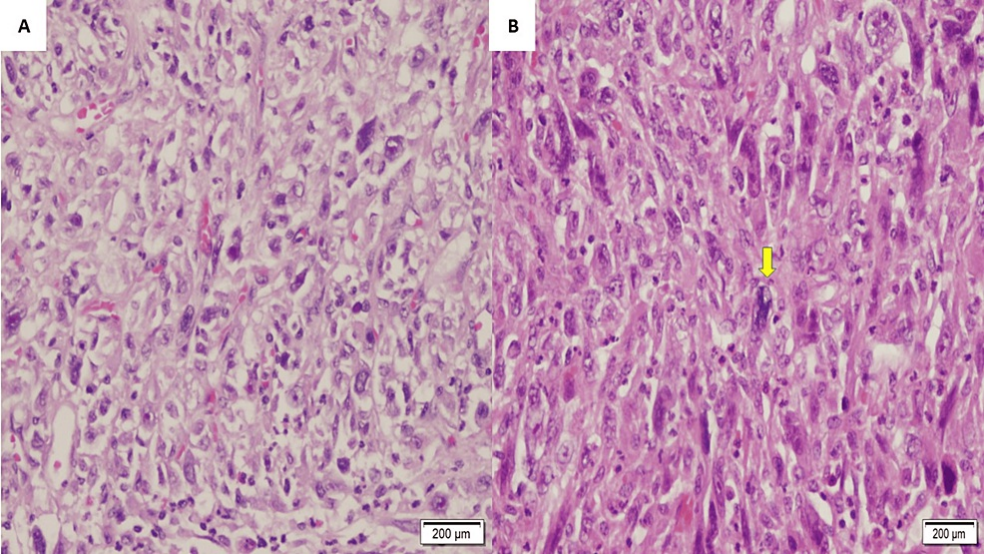
Histopathology examination by hematoxylin and eosin stain (H&E). (A): Tumor is composed of destructive sheets of non-cohesive growth neoplasm (H&E; 10x). (B): The tumor cells are typically large and display varying degrees of pleomorphism, often resembling mature histiocytes, note the atypical mitosis (yellow arrow) (H&E; 10x).

**Figure 4:**
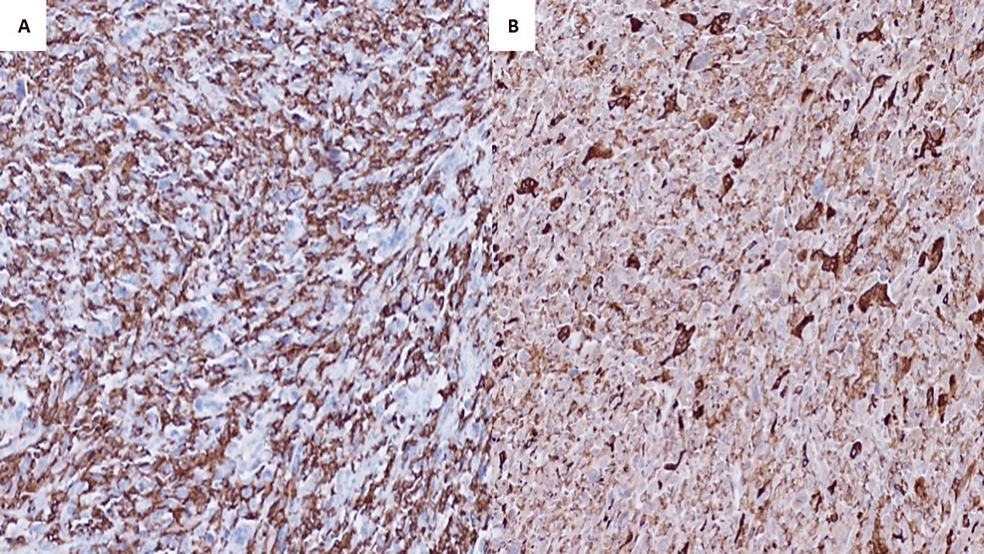
(A): Immunohistochemistry studies for CD163 show diffuse cytoplasmic immunoreactivity in histiocytic sarcoma (40x). (B): CD68 shows positive expression in the neoplastic cells (40x).

Gross pathology examination of the mass depicted a well-defined mass measuring 15 x 13 x 8 cm with a pink-tan heterogeneous predominantly solid cut surface with a central area of hemorrhage and necrosis (Figure 2). Microscopic examination showed a well-defined neoplastic growth arranged in sheets and fascicles (Figure 3). The neoplastic cells displayed a range of shapes, including spindle, round, ovoid, and giant cells with abundant eosinophilic to vacuolated cytoplasm containing very pleomorphic and hyperchromatic nuclei with prominent nucleolus. Atypical mitotic figures and necrosis were seen frequently. In addition, there was a significant presence of neutrophils infiltrating the area. A panel of immunohistochemical markers (IHC) was assayed. Neoplastic cells were positive for CD45, CD4, CD68, CD163, and vimentin while being focally positive for CD45Ro, CD43, and fascin (Figure 4). Assays were negative for pan-cytokeratin, P63, CK5/6, HMB-45, EMA, SMA, MSA, H-caldesmin, CD3, CD5, CD20, CD23, CD34, CD15, CD30, S100, Langerin, CD1a, CD35, myloidpreixodase (MPO), lysosome, and ALK-1.

The morphology along with the immunophenotype picture was diagnostic for HS. Surgical resection margins were negative. The patient was further discussed at MDTB and a decision was made to proceed with adjuvant chemotherapy. She was advised to get chemotherapy, but she refused initially and then developed an aggressive metastatic pattern. Unfortunately, the patient passed away from metastasis before her first-year follow-up.

## Discussion

HS is a rare hematolymphoid neoplasm that is often confused with other malignant neoplasms due to overlapping cytomorphological features. HS is derived from cells such as histiocytes and non-Langerhans cells. The term HS has been used since the 1970s when Mathé and colleagues described 110 cases of reticulosarcoma based on their histological and cytological findings [7]. According to the fifth edition of the World Health Organization (WHO) classification of haematolymphoid tumors, HS is categorized under the histiocytic/dendritic cell neoplasms group [8]. Histiocytic/dendritic cell neoplasms are classified after myeloid neoplasms because they originate from the same myeloid progenitors that generate cells belonging to monocytic cellular histiocytic/dendritic cell origin [9].

According to the literature review, HS can develop in the lymph nodes, and other areas apart from the lymph nodes, especially in the gastrointestinal tract and skin. This can result in severe illnesses and demonstrate aggressive clinical behavior. It is common for cases primarily originating at extranodal sites to be undetected and misdiagnosed. Connective tissue, respiratory system, and central nervous system are among the other areas that may be impacted [10]. After conducting a thorough search of the existing literature, it was discovered that only three previous instances of primary breast HS involvement have been documented [11-13] (Table 1).

**Table 1:** Summary of reported histiocytic sarcoma (HS) cases primarily involving the breast. CT: computed tomography; (FDG) PET-CT: positron-emission tomography/computed tomography using deoxy-2-[18F] fluoro-D-glucose; BM: bone marrow; CHOP: cyclophosphamide, doxorubicin, vincristine, prednisolone.

Author (Reference)	Age (years)/Sex	Clinical presentation	Tumor size	Hospital work-up	Treatment	Follow-up (months)
Nangal et al., 2014 [11]	40/Female	Left axillary mass in close proximity to the left breast, gradually increased for 3 months	13x11 cm	CT scans of thorax, and abdomen negative; BM negative	Surgical excision	Planned to receive 6 cycles of CHOP. No follow-up data available
Bang et al., 2019 [12]	75/Female	Palpable solitary mass in the right breast	1.4 cm	Whole body (FDG) PET-CT is negative	Surgical excision. No chemotherapy received.	No recurrence or metastasis/8 months
Higuchi et al., 2023 [13]	81/Female	Palpable mass in right upper breast for three months	3.5 cm	No cervical/axillary lymphadenopathies	Surgical excision. No chemotherapy received.	No recurrence or metastasis/50 months
Current case	40/Female	Progressively growing mass for one year in right breast	15x13x8 cm	CT scan of chest, abdomen & pelvis are negative	Surgical excision and planned for chemotherapy	Died due to tumor metastasis

Histopathology examination with the aid of immunohistochemistry is essential to confirm the diagnosis due to the lack of specificity in radiology studies. In the present case, a tissue biopsy was necessary to establish a conclusive diagnosis. The initial needle core biopsy alone could not serve as a definitive diagnostic tool. When examining non-epithelial tissue from a breast mass, it is crucial to consider the potential of HS as a viable alternative diagnosis. Tumor cells are generally large and exhibit variable pleomorphism, but they frequently resemble mature histiocytes. Cytomorphology evaluation of tumor cells typically reveals the presence of large, atypical cells with abundant eosinophilic cytoplasm, arranged in a diffuse sheet of non-cohesive proliferation. Additionally, there may be varying levels of inflammatory cells present in the tumor cells. Multinucleated giant atypical cells, erythrophagocytosis, or focal spindle cells can be seen. Atypical mitotic figures, apoptosis, and tumor necrosis can be easily identified in these cases. HS is categorized by the presence of one or more histiocytic markers, including CD163, CD68, and lysozyme. However, it does not have Langerhans cell expression such as CD1a and langerin. CD21, CD23, and CD35 found in follicular dendritic cells should be negative. Furthermore, myeloid lineage tests revealed no presence of CD13 and MPO [14]. These tumors are consistently negative for epithelial markers such as pan-cytokeratin and melanocytic markers such as HMB-45 and Melan-A. S100 protein expression may be present; it is typically weak and focally expressed. Other markers that can be expressed in HS include CD4, CD43, CD14, CD15, CD11c, MAC387, and HLA-DR [13,14].

There is an absence of positivity observed, as indicated by the absence of specific markers in B-cells and T-cells. The Ki-67 varies and there is currently no established standard for its proliferation index. It can be challenging to obtain a definitive diagnosis for histiocytic sarcoma, but it is important to also eliminate the possibility of other differential diagnoses which include poorly differentiated carcinomas, melanomas, sarcoma, diffuse large B-cell lymphoma, anaplastic large cell lymphoma, myeloid sarcoma, and other dendritic cell neoplasms [12].

There is currently no established standard treatment for primary breast HS [13]. In the present case, the patient was scheduled to receive adjuvant chemotherapy along with surgical resection. In an earlier case, one patient was scheduled to undergo a six-cycle CHOP treatment following surgical resection. Unfortunately, there is no available data regarding their follow-up progress [11]. Two other cases had been treated exclusively with surgical resection, without receiving any chemotherapy. Both patients experienced no recurrence or distant metastasis during the overall follow-up period of eight and 50 months, respectively [12,13]. Because this condition is rare and there are only a few case reports available in the area, it is difficult to gather substantial evidence. As a result, these cases are of great clinical significance.

## Conclusions

We reported a rare case where HS primarily appears in the breast parenchymal tissue of a 40-year-old female patient. Although diagnosing HS can be difficult, pathologists should be aware of it as a possible alternative diagnosis when handling non-epithelial breast tumors. Histopathology along with confirmatory immunohistochemistry markers are essential for definitive diagnosis. To the best of our knowledge, the current medical literature on HS only contains a few instances where breast involvement has been recorded. Additional studies are required to determine the appropriate treatment for patients diagnosed with primary breast HS.
